# The Association of Increased Oxidative Stress and Tumor Biomarkers Related to Polyaromatic Hydrocarbons Exposure for Different Occupational Workers in Makkah, Saudi Arabia

**DOI:** 10.7759/cureus.32981

**Published:** 2022-12-26

**Authors:** Heba M Adly, Saleh A Saleh

**Affiliations:** 1 Community Medicine and Pilgrims Health, Faculty of Medicine, Umm Al Qura University, Makkah, SAU; 2 Biochemistry, Faculty of Medicine, Umm Al Qura University, Makkah, SAU

**Keywords:** p21, p53, tumor markers, oxidative stress, exposure, polyaromatic hydrocarbons

## Abstract

Introduction: Exposure to occupational polyaromatic hydrocarbons (PAHs) is correlated with several adverse effects on human health, including bladder, lung, and skin cancer. The correlation between PAH exposure and oxidative stress and tumor markers needs to be further explored. Therefore, we conducted this study to examine the effect of acute exposure to PAHs on oxidative stress and tumor marker levels in occupational workers during the Hajj season in Makkah.

Methods: We conducted a cross-sectional study of 105 workers during Hajj; 60 workers were employed in the open air for ≥eight hours/day, exposed them to high levels of considerable traffic and huge crowds, and 45 workers served as our control group who were unexposed and working in a rural area. Using high-performance liquid chromatography, we analyzed participants' urinary 1-hydroxypyrene to determine PAH levels. Oxidative stress markers malondialdehyde (MDA), glutathione S-transferase (GST), and lactate dehydrogenase (LDH) were analyzed in serum using a spectrophotometer. The serum p53 and p21 proteins were analyzed using an enzyme-linked immunosorbent assay. We used IBM SPSS Statistics for Windows, Version 21.0 (IBM Corp., Armonk, NY, USA) to calculate multivariate logistic regression analysis for oxidative stress and tumor markers such as age, working period, and smoking status risk factors. Additionally, we evaluated associations between oxidative stress and tumor markers.

Results: The mean levels of MDA, GST, and LDH were significantly elevated in exposed workers compared to the control group (p<.001). Also, p53 and p21 protein levels were significantly higher in the occupationally exposed group than in the unexposed control group (p<0.05). No significant correlation between age and increased levels of p53 and p21 was found.

Conclusions: In our study, PAH exposure is significantly correlated with higher levels of oxidative stress and tumor marker levels in occupational workers. The evaluation of oxidative stress and tumor marker indicators can efficiently identify workers at high risk of PAH exposure and may assist in preventing future health concerns. More biomarkers should be included in other longitudinal studies to address exposure related to different health risks among workers, especially cancer risk. More prospective studies are required to validate diagnostic utilities and efficiencies of different biomarker combinations.

## Introduction

Adverse health effects, such as hepatorenal toxicity, cardiopulmonary disease, and several cancers, have been correlated with work-related and environmental exposures to agents containing polyaromatic hydrocarbons (PAHs). The main sources include tobacco smoking, waste burning, residential, manufacturing, heating, and engine gases. Individual exposure to PAH involves breathing contaminated air, nutritional consumption, and dermal contact [1]. Oxidative stress markers have a documented positive correlation with high exposure to PAHs in pregnant women [2], infants, and industrial workers [3]. However, the studies on this association in healthy young adults are inadequate [4]. The correlation between exposure among occupational workers, cellular oxidative insults, and multiple organ dysfunctions is doubtful [5].

Oxidative stress is produced by the discrepancy between the production of reactive oxygen species (ROS) and antioxidant defenses in the human body. It may influence the correlation between exposure to PAHs and metals and their harmful health effects. Urinary PAH concentrations have been positively correlated with serum biomarkers of inflammation and oxidative stress, including C-reactive protein (CRP) and gamma-glutamyl transferase (GGT) [6]. Urinary concentrations of 1-hydroxypyrene (1-OHP), 1-hydroxynaphthalene, 2-hydroxynaphthalene, 2-hydroxyfluorene, and 9-phenanthrenen have been used as biomarkers to measure PAH exposure [7]. This study aimed to assess the effects of acute exposure to PAHs on oxidative stress and tumor marker levels in occupational workers during the Hajj season in Makkah.

## Materials and methods

Study design and sample selection

Sixty people were included in the study. The study population comprised workers who work with pilgrims throughout the Hajj, such as truck and lorry drivers and pilgrim assistance personnel. All workers included in the study are employed in the open air for eight or more hours/day, exposing them to considerable traffic and huge crowds. The control group consisted of 45 non-exposed workers in an indoor environment within the same study period, primarily in rural areas.

Experienced investigators supervised the surveys as open-ended and multiple choice questions represented to study participants while interviewing and getting their consent to collect the required demographic and socioeconomic data comprising age, occupation time, and smoking habits. We considered people who had smoked for three months to be included in the study as smokers. All participants provided written informed consent to participate in the study. The study excluded people exposed to well-known mutagenic agents, such as radiation therapy and chemotherapy, in the past three months.

The study protocol was certified by the Ethics Review Board for Human Studies at the Faculty of Medicine, Umm Al-Qura University (Approval No. HAPO-02-K-012-2020-07-413 in accordance with the Saudi National Committee for Bioethics HABO-02-K-012).

Sampling of PAHs in ambient air

PAH sampling was conducted using a mini volume sampler (Airmetrics, Springfield, OR, USA) to collect PM10 air sampling. The air sampling unit was positioned at a 10-m height with a 16.6 L/min flow rate, set on a 47-mm Teflon filter for 24 hours, and accumulated weekly according to the United States Environmental Protection Agency standard method (EPA/625/R-96/010b) [8]. PM10 filters were weighed and acclimated at 35-40°C with 60% to 70% humidity.

PAH laboratory analysis

Filter samples were dried and reweighed to determine PM10 concentrations and kept in dark conditions to be ready for analysis. PAHs were analyzed using gas chromatography/mass spectrometer ([GC/MS], Clarus 600; PerkinElmer, Waltham, MA, USA) and separated with 10 ml of dichloromethane/n-hexane (1:1), fractionated by column chromatography, and diluted by 20 ml of n-hexane/dichloromethane (1:1, v:v). A 2-μl sample of the extract was analyzed on the GC/MS. This GC was calibrated with a diluted standard solution of different PAH compounds (Supelco, Inc., Bellefonte, PA, USA).

Determination of 1-OHP in urine

1-OHP in urine was analyzed by high-performance liquid chromatography (HPLC) using 10-ml urine samples deconjugated enzymatically and prepared in C18 octadecyl cartridges. Samples were washed with 10 ml of water and eluted with 9 ml of methanol. The elute components were separated over HPLC (HPLC 300; PerkinElmer) using a 150 x 4-mm I.D. LiChrosorb RP-18 column. The column temperature was adjusted to 40°C with a flow rate of 0.8 ml/min. The solvent gradient was as follows: 5 minutes of methanol-water (46:54); a linear gradient in 35 minutes with methanol-water (94:6); hold for 10 minutes. 1-OHP was assayed with 242-nm excitation and 368-nm emission wavelengths, paired with SimplicityChrom™ CDS software (PerkinElmer). 1-OHP concentrations were standardized to urinary creatinine. Urinary creatinine concentrations were analyzed using a standard colorimetric method resulting from the picric acid reaction and absorbed at 520 nm.

Serum oxidative stress markers

We measured the concentrations of serum lipid peroxidation products as the thiobarbituric acid adduct of malondialdehyde (MDA) spectrophotometrically HumaStar 300SR (HUMAN Gesellschaft für Biochemica und Diagnostica mbH, Wiesbaden, Germany) using HUMAN Clinical Chemistry Reagents per the manufacturer's guidelines. The MDA concentration was measured with a sensitivity between 0.025 and 6.25 μg/mL. The reaction mixture consisted of 0.1 M Na phosphate (pH 6.5), 30 mM GSH, 30 mM 1-chloro-2,6-dinitrobenzene, and serum. The optical density was determined with absorbance at 340 nm. Lactate dehydrogenase (LDH) assay terms used HumaStar 300SR with a measuring range of 15 to 1600 U/l; 0.25 to 26.7 µkat/l and a reference range of 225 to 450 U/l (3.75 to 7.50 µkat/l).

Serum tumor marker measurement

Blood samples were collected from the participant at the end of their work shift using plain tubes and allowed to clot. Blood sample tubes were centrifuged; the serum was separated from the samples, refrigerated at -20°C, and analyzed for one week. All participants' serum levels of p53 protein were analyzed in serum samples using Human p53 enzyme-linked immunosorbent assay (ELISA) kits (Sigma-Aldrich, St. Louis, MO, USA) based on the manufacturer's guidelines. Serum levels of p21 protein were investigated via the Human P21 ELISA Kit (Proteintech Group Inc., Rosemont, IL, USA).

Statistical analysis

We used IBM SPSS Statistics for Windows, version 21.0 (IBM Corp., Armonk, NY, USA) to analyze our data. Statistics were defined as mean ± standard deviation. We used multivariate logistic regression analysis for oxidative stress and tumor markers as age, working period, and smoking status risk factors. 1-OHP concentrations indicated PAH exposure levels. Additionally, associations between oxidative stress and tumor markers were evaluated. All statistical tests were two-sided, and p<0.05 was considered significant.

## Results

Patient demographic characteristics

Table 1 presents the demographic data of occupationally exposed workers and the unexposed control group. Age, working period, smoking status, and 1-OHP were compared between controls and exposed workers working in different occupations and areas in Makkah. The mean age was 34.5 ± 4.2 years for the exposed group and 36.5 ± 5.1 years for the unexposed group. We found no statistical differences in age or employment periods (p>0.05).

**Table 1:** Summary data for study population SD, standard deviation; 1-OHP, 1-hydroxypyrene

Population Variables	Occupationally Exposed Group (n=60)	Unexposed Control Group (n=45)	P-value
	Mean ± SD	Mean ± SD	
Mean Age (years) ± SD	34.5 ± 4.2	36.5 ± 5.1	>0.05
Mean Employment Period (years) ± SD	15.6 ± 2.65	10.93 ± 3.53	>0.05
Current smokers (Yes, n, %)	41 (68.33%)	26 (57.7%)	>0.05
1-OHP (μmol/mol creatinine)	4.85 ± 0.78	17.98 ± 3.09	< .001>

Total PAH levels in ambient air

The total concentrations of PAH ranged from 20.67 to 34.7 ng/m3 in individual working areas with relatively high road traffic throughout the sampling period. The US National Institute for Occupational Safety and Health has advised that the maximum level for PAHs be set at the lowest measurable concentration (0.1 mg/m3) for indoor exposure, while outdoor ambient air has a maximum acceptable PAH level of 10 mg/m3 [9].

Serum oxidative stress and tumor markers

**Figure 1:**
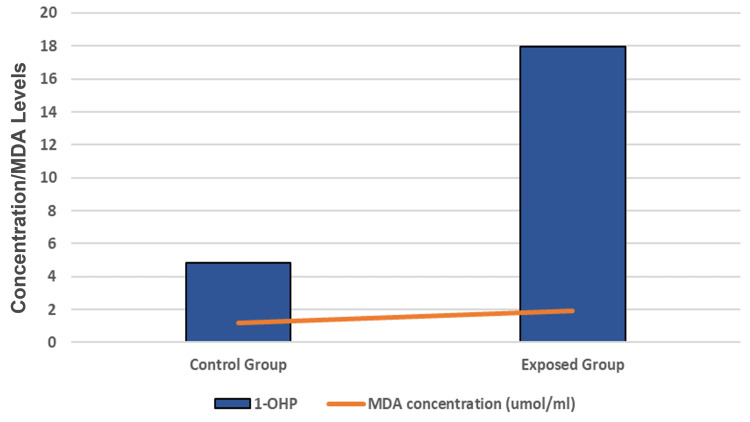
Correlation of urinary 1-OHP concentration and levels of MDA. The differences between the control group (unexposed) and the occupational group (exposed) were significant (p<0.001). 1-OHP is measured as μmol/mol creatinine and MDH is measured as µmol/ml. 1-OHP, 1-hydroxypyrene; MDA, malondialdehyde

**Figure 2:**
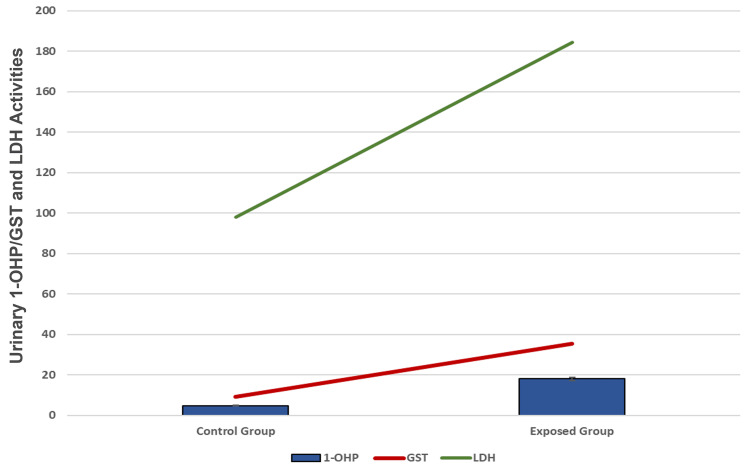
Correlation of urinary 1-OHP and levels of GST and LDH activities in serum. The differences between the control group (unexposed) and the occupational group (exposed) were significant (p<0.001). 1-OHP is measured as μmol/mol creatinine, GST and LDH activities are measured as U/ml. 1-OHP, 1-hydroxypyrene; GST, glutathione S-transferase; LDH, lactate dehydrogenase

Figure 1 and Figure 2 indicate the distribution of oxidative stress and tumor markers in all participants correlated with urinary 1-OHP concentrations. Mean MDA, glutathione S-transferase (GST), and LDH levels were significantly higher in exposed workers than in the control group (p<.001). Mean serum levels of p53 and p21 proteins were significantly higher in the occupationally exposed group than in the unexposed group (p<0.05) (Table 2).

**Table 2:** Mean serum p53 and p21 protein levels SD, standard deviation.

Serum Protein	Occupationally Exposed Group (n=60)	Unexposed Control Group (n=45)	P-value
Mean p53 (ng/ml) ± SD	2.2 ± 0.5	1.7 ± 0.3	>0.05
Mean p21 (ng/ml) ± SD	1.4 ± 0.3	1.1 ± 0.2	>0.05

Results of the associated variables of the study groups on oxidative stress and tumor markers

Multivariate regression evaluations with variation for potential parameters (age, employment period, smoking status, 1-OHP concentration) were produced to evaluate the possible determining factors of increased oxidative stress and tumor marker concentrations. We used the odds ratio (OR) of age, employment period, smoking status, and level of 1-OHP to evaluate their effect on the biomarkers studied. The correlation between age with increased risk of p53 and p21, smoking with an increase in the risk of GST activities, and the risk of MDA concentrations was not significant (p=0.055, p=0.057, respectively; Table 2, Table 3). The OR and confidence intervals (CI) of smoking were significantly correlated with MDA (p=0.004, OR=14.07; 95% CI, 2.30 to 85.93), p53 (p=0.004, OR=5.76, 95% CI, 1.78 to 18.66), and p21 (p<.001, OR=5.98, 95% CI, 2.57 to 13.87). PAH exposure was associated with a significantly (p<.001) high risk of high levels of oxidative stress and tumor markers p53 and p21. Associations of oxidative stress and tumor markers for all studied groups are summarized in Table 3. There was a positive and significant correlation between oxidative stress and tumor markers. Differences were significant (p<0.0001).

**Table 3:** Oxidative stress and tumor variables in the exposed group MDA, malondialdehyde; GST, glutathione S-transferase; LDH, lactate dehydrogenase

Serum Protein	MDA		GST		LDH	
	r	P-value	r	P-value	r	P-value
p53	0.972	<0.0001	0.945	<0.0001	0.912	<0.0001
p21	0.728	<0.0001	0.852	<0.0001	0.607	<0.0001

## Discussion

Makkah has approximately 1,700,000 residents, but throughout the Hajj period, the city has hosted 2,371,675 visitors, 75% from outside the country. During the Umrah period, Makkah hosts nearly 3,000,000 visitors [10]. Accordingly, a remarkable increase in public transportation involving automobiles directly increases fuel consumption and causes elevated levels of air pollution consisting of dust, metals, and PAHs. Approximately 287,300 workers provide local services to pilgrims; many of them work outdoors and are highly subjected to acute pollution, and about 18,400 buses and shuttle buses were transported during Hajj to commute pilgrims around Makkah [10].

Our study examined the impact of exposure to PAHs on oxidative stress and tumor biomarkers among occupationally exposed workers and control workers. We also examined potential correlations among workers' traits linked to oxidative stress and tumor biomarkers. Earlier studies only assessed the impact of PAHs and heavy metals on oxidative stress levels, and the results were inadequate and contradictory [5]. A study of pregnant women reported that urinary hydroxylated PAHs and metal mixes were positively correlated with 8-hydroxyguanosine (8-OHdG) but not in the same way with 8-iso-prostaglandin F2α [5]. PAHs have shown metabolic stimulation to form free radical mediates that endure redox cycling to produce ROS, which is bound covalently to biomolecules such as lipids, proteins, and DNA, leading to lipid peroxidation, the creation of protein and DNA adducts, oxidative stress, and DNA alterations. These processes have been associated with PAH and lead to immunologic, renal, and lung dysfunctions. Exposure to PAH has been correlated with high lipid peroxidation product levels in plasma, oxidative stress biomarkers, oxidative DNA damage, and failure in lung function [11].

In our study, exposed workers had substantially high PAH concentrations as urinary 1-OHP. Our results were similar to another study performed in Thailand of 60 workers and 45 control subjects. That study found elevated levels of oxidative stress and tumor markers induced by PAH exposure [12]. In Korea, another study stated that outdoor time had a minimal positive correlation with urinary 1-OHP levels in non-occupationally exposed people [13].

Similarly, MDA and LDH levels were significantly higher in the serum of exposed occupational workers than in controls. In a cross-sectional study from 2003-2008 in the United States of America of 660 youths aged 12 to 19, researchers found statistically significant associations between PAH metabolites and serum concentrations of MDA, GGT, CRP, estimated glomerular filtration rate, and uric acid [14]. A study in northern China reported that PAHs were associated with dose-dependent DNA damage, micronuclei rate, MDA levels, and LDH activity [15].

According to our results, PAH significantly activates GST activity. GST provides a phase II enzyme in detoxifying xenobiotics and maintaining intracellular redox balance in the existence of its substrates. Consequently, the increase of GST activity in exposed workers could represent an adaptation or protection opposed to the burden of mutagens and carcinogens in humans. The increases in MDA, LDH, and GST were associated with the concentration of urinary 1-OHP, depending on working environments and occupation time. Elevated concentrations of 1-OHP could reveal greater dose exposure or faster detoxification activity.

Furthermore, smoking also altered MDA (p=0.004, OR=14.07, 95% CI, 2.30 to 85.93). Smoking induces similar effects in GST activities (p=0.048, OR=2.57, 95% CI, 1.01 to 6.57). Various studies demonstrated that MDA levels were higher among smokers regardless of the degree of smoking behavior [16]. Our findings also align with a Nigerian study that showed positive correlations between 1-OHP (r=0.570, p=0.000), oxidative stress index (r=0.299, p=0.035), and MDA (r=-0.265, p=0.008) in barbecue workers [17].

Substantial increases in tumor markers p53 and p21 were noted in exposed occupational workers compared to unexposed controls. These results agree with results from another study conducted in Makkah within the same period evaluating different types of PAHs such as benzo[a]pyrene, dibenzo[a,l]pyrene, benzo[a]anthracene, dibenzo[a,h]anthracene, benzo[b]fluoranthene, and indeno[1,2,3-c,d]pyrene. These PAHs were measured in contaminated ambient air and ranged from 6.34 to 37.4 ng/m3, and tumor markers levels were measured in blood serum [18]. 

p53 is active in microRNA regulation and promotes several biofunctions within cells. Despite these carcinogenic properties, it acts as a tumor-suppressing protein by regulating various reactions, such as genotoxic cellular stresses, leading to stimulation of DNA restoration, initiation of cell cycle arrest, and apoptosis [19]. The mutation of the p53 gene changes its ability to promote tumor-suppressing pathways, leading to tumorigenesis. p53 plays a vital role in maintaining genomic stability and homeostasis. The p53 protein regulates the appearance of its downstream effector genes, whose expressions are related to critical cellular actions such as DNA repair, cell cycle control, and apoptosis. Overexpression of the p53 protein is a cellular reaction to genotoxic stress [20].

Our results show that PAHs increase serum tumor markers that can stimulate tumor origination. This could help distinguish potential early-stage lung cancer from benign masses [21]. Our results were consistent with a study conducted in the Czech Republic among police officers in different cities, where p53 plasma levels were positively associated with PAH exposure [22]. Moreover, chronic exposure to PAH disturbed the cellular DNA damage reaction, producing S-phase arrest and preventing apoptosis. This dysregulation of the PAH metabolic rate and the response to DNA damage changed the cellular homeostasis and increased cell sensitivity to successive PAH exposures, which increases the possibility of genomic metamorphosis and instability [23]. More than half of our exposed group (67.53%) and more than half of our control group (58.33%) were smokers, and they had significantly elevated P53 and p21 concentrations (p>0.05). This increase in p53 and p21 is similar to a study that verified macrophage production of interleukin 1β (IL-1β) and tumor necrosis factor-alpha (TNF-α) [24] and reported that smoking and contact with smoking increased TNF-α levels in cigarette-smoke-induced pulmonary vasculature damage [19].

p53 plays a significant role in ruling intracellular redox homeostasis. Oxidative stress and p53 stimulation are frequent reactions to chemical exposure and might play crucial roles in chemical-induced poisoning. Stimulating p53 can apply either pro-oxidant or antioxidant activity, depending on the context [25]. We identified a positive and significant correlation between oxidative stress and tumor markers. Oxidative stress plays vital functions in the pathogenesis of various diseases, including elderly patients and those with progressive illness and cancer. These combined observations indicate that oxidizing forms lead to DNA damage which consequently facilitates a p53-dependent response such as cell cycle arrest and apoptosis. Also, hydrogen peroxide stimulates signaling on stress-activated protein kinases, c-Jun N-terminal kinases (JNKs), and p38 mitogen-activated protein kinases may activate p53 [26].

Our study had several significant limitations. First, increased serum tumor markers such as p53 and p21 are usually present in an advanced tumor stage. Second, oxidative stress differs according to age, the oxidative stress hypothesis of aging assumes that age-associated functional losses are due to the accumulation of reactive oxygen and nitrogen species-induced damage [27]. At the same time, oxidative stress is involved in several age-related illnesses, such as cardiovascular disease, chronic obstructive pulmonary disease, neurodegenerative diseases, chronic kidney disease, and cancer [28].

## Conclusions

We conducted this study to assess the effect of acute exposure to PAHs on oxidative stress and tumor marker levels in some occupational workers during the Hajj season in Makkah. PAH exposure is significantly correlated with higher levels of oxidative stress and tumor marker levels in occupational workers. Biomarkers can serve as vital short-term markers for different health adverse especially in reference to cancer for occupational workers. More studies are required to validate their diagnostic utilities. Therefore, assessing these indicators can effectively predict and protect workers from future health. 
